# Left atrial activation and asymmetric anatomical remodeling in patients with atrial fibrillation: The relation between anatomy and function

**DOI:** 10.1002/clc.23515

**Published:** 2020-11-17

**Authors:** Sotirios Nedios, Susanne Löbe, Helge Knopp, Timm Seewöster, Jordi Heijman, Harry J. G. M. Crijns, Arash Arya, Andreas Bollmann, Gerhard Hindricks, Borislav Dinov

**Affiliations:** ^1^ Department of Electrophysiology Heart Center at University of Leipzig Leipzig Germany; ^2^ Cardiac Arrhythmia Service Massachusetts General Hospital Boston Massachusetts USA; ^3^ Department of Cardiology, Cardiovascular Research Institute Maastricht (CARIM) Maastricht University Medical Center Maastricht The Netherlands

**Keywords:** asymmetry, asynchrony, atrial fibrillation, atrial remodeling, diastolic dysfunction, pulsed‐wave tissue Doppler imaging

## Abstract

**Background:**

Identifying patients with advanced left atrial (LA) remodeling before catheter ablation (CA) of atrial fibrillation (AF) is crucial.

**Hypothesis:**

This study aimed to identify echocardiographic parameters associated with changes in anatomy and conduction properties of the left atrium (LA).

**Methods:**

We examined 75 AF patients prior to CA and measured the intervals from the P‐wave‐onset to four mitral annulus sites by pulsed‐wave tissue Doppler imaging (PW‐TDI). Patients were grouped to an upward U‐pattern (delayed anterior activation) and a downward D‐pattern (earliest LA activation anterior). CT‐data were used to measure the LA volume (LAV). LAV was divided into anterior‐ (LA‐A) and posterior‐parts by a plane, parallel to the posterior wall and between the veins and the appendage, to calculate the asymmetry index (ASI = LA‐A/LAV).

**Results:**

Patients with U‐pattern (n = 66) had a higher ASI (65 ± 6 vs. 61 ± 3%, *p =* .014), older age (61 ± 11 vs. 51 ± 11 years, *p =* .03) and more diastolic dysfunction (71 vs. 22%, *p =* .008) Multivariate regression showed that age (OR 1.1 per year, CI 1.007–1.199) and diastolic dysfunction (OR 6.36, CI 1.132–35.7, *p =* .036) were independent predictors of the U‐pattern. Diastolic dysfunction (B 4.49, CI 1.61–7.37, *p =* .003) was the only independent predictor of ASI in linear regression analysis.

**Conclusion:**

AF patients with a U‐pattern have an increased LA asymmetry. Diastolic dysfunction is a common cause of this LA activation and remodeling. Therefore, detection of a U‐pattern signifies patients with advanced AF and may facilitate selection for an appropriate ablation strategy.

**Abbreviations**
AF: atrial fibrillation
ASI: asymmetry index
CA: catheter ablation
CS: coronary sinus
CT: computed tomography
LA: left atrium
LA‐A: left atrial anterior (LA‐A) partial volume
LAA: left atrial appendage
LA‐P: left atrial posterior (LA‐P) partial volume
LAV: left atrial volume with anterior (LA‐A) and posterior (LA‐P) partial volumes
LV: left ventricle
LVDD: left ventricular diastolic dysfunction
LV‐EF: left ventricular ejection fraction
MA: mitral annulus
PV: pulmonary vein
PW: pulsed‐wave
TDI: tissue Doppler imaging
U: upward (activation pattern)

## 1 INTRODUCTION

Atrial fibrillation (AF) is associated with left atrial (LA) remodeling, characterized not only by dilatation but also by changes of LA symmetry. This is particularly true for greater LAs, when due to anatomical constrictions LA extension occurs nonuniformly. This asymmetric LA dilatation is a strong predictor of poor outcome after catheter ablation (CA).^1^, ^2^, ^3^

On the other hand, the shortening of the atrial refractory period and the slowing of intra‐atrial conduction contribute to AF perpetuation.^4^, ^5^ Several studies have shown that intra‐atrial delay is also associated with AF progression and poor outcomes after CA or cardioversion.^6^, ^7^ Recently, we proposed a noninvasive method to evaluate LA activation and asynchrony using pulsed‐wave tissue Doppler imaging (PW‐TDI). We found a specific activation pattern with earlier inferior LA activation, and delayed anterior LA activation, which we called U (upward) pattern to be more common among AF patients.^8^ Previous studies have shown that conduction time at the lateral mitral annulus (MA) is a significant predictor of AF recurrence after CA.^6^ In contrast, we examined LA activation at four sites and found that LA activation indexes were significantly associated with abnormal LA voltage and recurrences after CA. The U‐pattern was more common in those with LA scar than those with normal LA voltage (100% vs. 84%, *p =* .015).^9^ Thus, we hypothesized that conduction disturbances in the Bachmann bundle (BB) or asymmetrical anatomical LA remodeling may explain these findings.

To further investigate the relation between electromechanical and anatomical remodeling, this study aimed to explore the differences in LA geometry according to the patterns of LA activation seen by PW‐TDI in AF patients.

## 2 METHODS

### 2.1 Patients

We prospectively studied a total of 200 patients from 2014 to 2015. All patients had documented AF and underwent CA with image‐integration using preprocedural computed tomography (CT). Of them, 75 patients were in sinus rhythm at the admission and were included in this study. Exclusion criteria were previous CA for arrhythmias, impaired left ventricular ejection fraction (LV‐EF), severe valvular disorders, pacemaker stimulation, or intraventricular conduction delay, overt pre‐excitation, history of palpitations without ECG documentation, and age <18 years. All patients gave written informed consent and data were collected in accordance with the Declaration of Helsinki and the institutional committee approved the study.

### 2.2 Imaging

All patients underwent a comprehensive transthoracic echocardiographic examination in sinus rhythm (Vivid 7, General Electric, Milwaukee, Wisconsin), according to the guidelines of the American Society of Echocardiography. The LA emptying fraction was calculated as (LAV max. – LAV min.)/LAV max. The 4‐chamber view LA area was indexed for body surface area. LV‐EF was measured by the Simpson method. Diastolic function (left ventricular diastolic dysfunction [LVDD]) was evaluated using pulsed‐wave (PW) Doppler recording of the mitral valve flow (E‐wave, A‐wave, E‐wave deceleration time). An apical 4‐chamber view was used to obtain longitudinal velocities (e') of both the medial and lateral MA. The ratio of transmitral diastolic peak velocity to the average MA diastolic peak velocity (E/e') was calculated. LVDD presence was defined according to current recommendations in case of E/A ≤ 0.8, DT > 200 ms, E/e' ≤ 8 (grade 1), 0.8 < E/A < 1.5, 160 ms < DT < 200 ms, and 9 < E/e' < 12 (grade 2) or E/A ≥ 2, DT < 160 ms, E/e' > 12 (grade 3).

**FIGURE 1:**
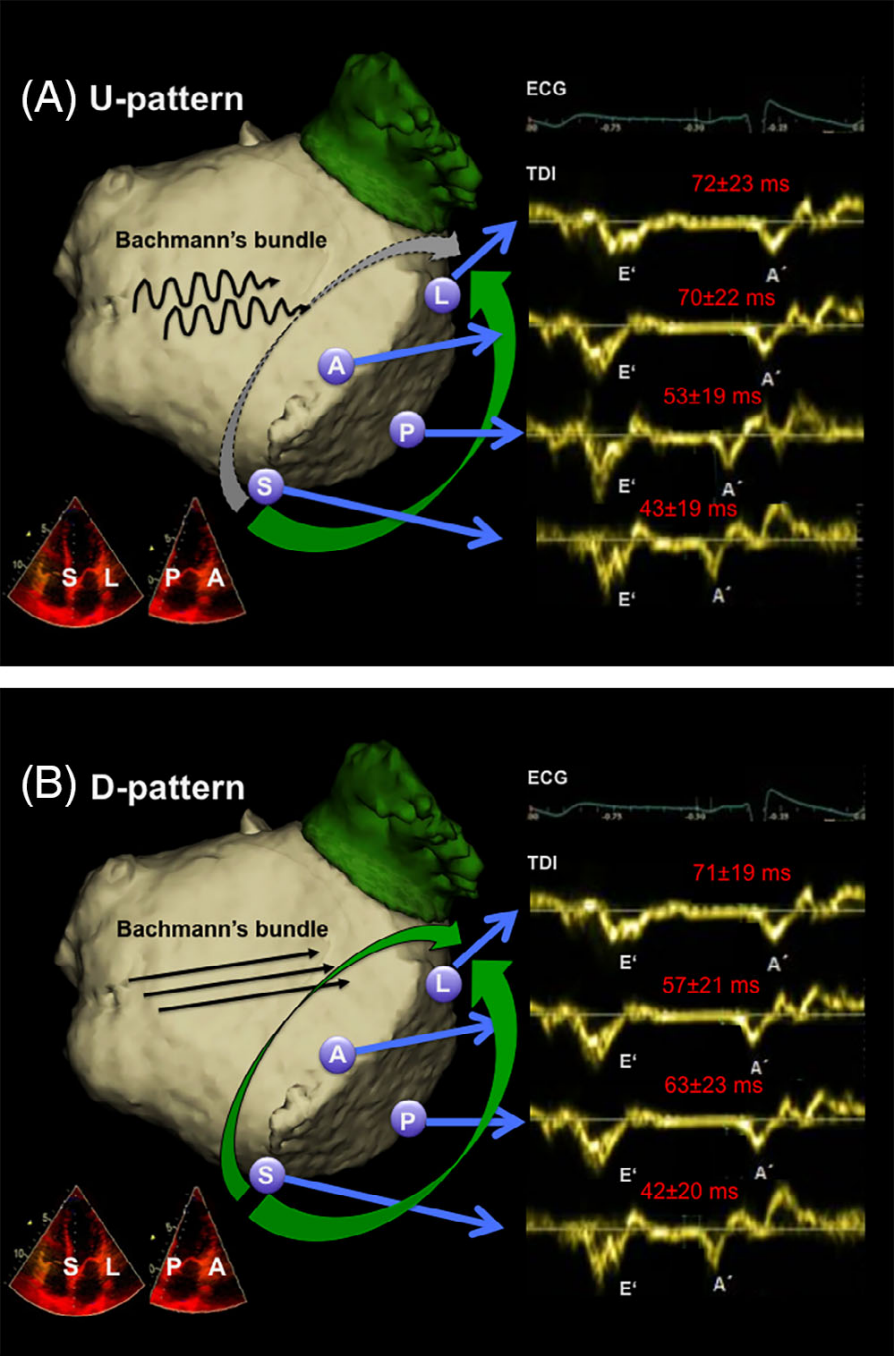
Left atrial (LA) activation patterns. An upward (U) activation pattern (1A) is characterized by slow Bachmann conduction resulting in longer anterior than posterior activation whereas a D‐pattern (1B) represents the normal (simultaneous anterior and posterior) activation of the mitral annulus as measured by tissue‐Doppler imaging (TDI). The numbers represent the mean interval from the P wave (ECG) to the mechanical activation of the septal (S), anterior (A), posterior (P) and lateral (L) part mitral annulus, measured by TDI on apical 4 and 2 chamber views (red)

PW‐TDI was used to sample the septal, lateral, anterior, and posterior LA above the MA. Time intervals (average of min. three cardiac cycles) from the onset of the P‐Wave to the onset of the local A' wave (P‐A') were measured at a 200 cm/sec. Patients with variable P wave morphologies or unclear P/A' onset were discarded. Measurement was blinded to other patient data. Based on our previous results, patients were categorized depending on the activation sequence around the MA in those with an upward LA activation (U‐pattern; delayed anterior and earlier posterior MA activation) and a downward D‐pattern (earlier activation at anterior MA, Figure 1).

**FIGURE 2:**
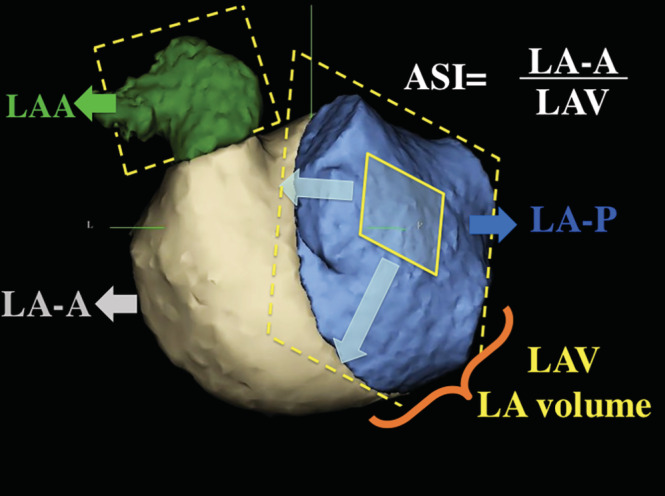
A cutting plane, between the pulmonary veins and the left atrial (LA) appendage (LAA) and parallel to the posterior wall, divides the LA volume (LAV) into anterior (LA‐A) and posterior (LA‐P) parts. The asymmetry index (ASI) is the ratio LA‐A/LAV

Cardiac‐CT was performed with a multidetector 64‐row helical system (Brilliance 64, Philips Medical Systems, Best, The Netherlands). Image acquisition was electrogram‐gated with 70–120 KV, 850 mAs, 0.6 mm beam collimation, 0.625–1.25 mm thickness, 20–30 cm field‐of‐view, and 90 ml of an iodinated contrast medium (Ultravist 370, Bayer Vital, Germany). End‐systolic data were used for 3D volume rendering (EnSite Verismo, SJM, Minnesota). The left atrial volume (LAV) was calculated after exclusion of the atrial appendage (LAA) and the pulmonary veins (PV). A cutting plane, between the PV ostia and the atrial appendage and parallel to the posterior wall, divided the LAV. The resulting anterior (LA‐A) and posterior (LA‐P) volumes were calculated and the ratio LA‐A/LAV × 100 was defined as the asymmetry index (ASI, % Figure 2). Analysis was blinded to other patient data. Intra/interobserver variability was assessed by measurement of 20 patients at baseline and 4 weeks later by two investigators in a blinded fashion.

### 2.3 Electrophysiological study and catheter ablation

An electrophysiological study was performed in all patients as previously described.^8^ A decapolar steerable catheter was inserted in the coronary sinus (CS). The time from the onset of the sinus P‐wave to local sharp CS signal was measured at CS poles 9–10 and 1–2. After transseptal access, heparin was used to achieve ACT > 300 seconds. A circumferential PV isolation was performed using irrigated catheters and an electroanatomical mapping system (Carto 3; Biosense Webster, Diamond bar, California or EnSite Velocity; Endocardial Solutions, St. Paul, Minnesota). Additional ablation was performed to connect low‐voltage areas (<0.5 mV) with electrically unexcitable landmarks.^10^

### 2.4 Statistics

Categorical variables are reported as frequencies and percentage. Continuous variables are expressed as mean and *SD*. Kolmogorov‐Smirnoff test was used to analyze the distribution of continuous variables. On that basis, parametric variables were compared by means of paired Student's *t*‐test (for two groups) and nonparametric variables by Wilcoxon‐test or *χ* ^2^ test. Intraobserver and interobserver variability was assessed with Pearson's *r*‐values. Clinical variables and imaging measurements were then evaluated with univariate regression analysis to determine their association with U‐pattern and ASI. Variables with *p <* .1 were included in a forward stepwise multivariate model to determine factors independently associated with U‐pattern. A two‐tailed *p*‐value less than .05 was considered statistically significant. Analysis was performed with SPSS 21.0 (SPSS Inc., Chicago).

## 3 RESULTS

### 3.1 Baseline characteristics

The majority of patients were males (55%) with a mean age of 59 ± 10 years and a normal LVEF (Table 1). The LV‐EF, the LV septum thickness, the LA diameter, and LA index did not differ significantly between the groups. The anterior P‐A' intervals were longer in the U‐pattern patients whereas the rest of the intervals were similar between the two groups. There was a significant correlation between the P‐A' at the lateral MA and the P‐CS (r = 0.67, *p <* .001) activation times.

**TABLE 1:** Comparison of atrial fibrillation (AF) patients with a U‐pattern and a D‐pattern, examined by pulsed‐wave tissue Doppler imaging (during sinus rhythm)

Baseline characteristics	All AF pts.	U‐pattern	D‐pattern	*p*‐value
Number, n	75	66	9	
Age, years	59 ± 10	61 ± 11	51 ± 11	.03
BMI, n	26 ± 5	26 ± 5	26 ± 6	.85
Female, n (%)	34 (45)	32 (49)	2 (22)	.71
Heart failure, n (%)	4 (5)	4 (6)	—	.13
Coronary/vascular disease, n (%)	6 (8)	4 (6)	2 (22)	.31
Diabetes, n (%)	10 (13)	9 (14)	1 (11)	.83
Hypertension, n (%)	55 (73)	47 (71)	8 (89)	.18
Persistent atrial fibrillation, n (%)	17 (23)	15 (23)	2 (22)	1.00
Beta‐blockers, n (%)	64 (85)	57 (66)	7 (78)	.50
Antiarrhythmic drugs Cl. I/III, n (%)	26 (35)	24 (36)	2 (22)	.39
Echocardiography				
LV ejection fraction, mm	64 ± 7	64 ± 6	63 ± 10	.84
Interventricular septum, mm	11 ± 2	11 ± 2	12 ± 2	.48
LV end diastolic diameter, mm	49 ± 6	49 ± 6	48 ± 4	.61
LA diameter, mm	41 ± 6	41 ± 6	41 ± 5	.95
LA area index, mm/m^2^	20 ± 3	20 ± 3	20 ± 4	.48
LA emptying fraction, %	49 ± 16	48 ± 17	58 ± 7	.22
E/A, n	1.3 ± 0.4	1.3 ± 0.4	1.0 ± 0.3	.08
Deceleration time, ms	228 ± 55	228 ± 58	225 ± 23	.79
Diastolic dysfunction, n (%)	45 (60)	43 (71)	2 (22)	.008
Mitral annulus P‐A' septal, ms	43 ± 19	43 ± 19	42 ± 20	.91
Mitral annulus P‐A' lateral, ms	72 ± 23	72 ± 23	71 ± 19	.88
Mitral annulus P‐A' posterior, ms	53 ± 19	52 ± 19	63 ± 23	.09
Mitral annulus P‐A' anterior, ms	70 ± 22	72 ± 21	57 ± 21	.049
P‐CS 9–10, ms	56 ± 20	56 ± 21	52 ± 7	.94
P‐CS 1–2, ms	82 ± 19	82 ± 20	82 ± 9	.64
Computed tomography				
LA volume, ml	102 ± 31	101 ± 32	109 ± 30	.47
LA anterior volume, ml	37 ± 13	36 ± 14	42 ± 10	.18
LA posterior volume, ml	65 ± 21	65 ± 21	66 ± 20	.83
Asymmetry index (ASI), %	64 ± 6	65 ± 6	61 ± 3	.01

Abbreviations: BMI, body mass index; CL., classm; LA, left atrial; LV, left ventricular.

### 3.2 Relation of the LA asymmetry and the U‐pattern with diastolic dysfunction

The intra and interobserver correlation coefficients were ≥0.90 as previously reported.^11^ AF patients with a U‐pattern (n = 66) had increased LA asymmetry (ASI 65 ± 6 vs. 61 ± 3%, *p =* .014), older age (61 ± 11 vs. 51 ± 11 years, *p =* .03) and more diastolic dysfunction (71 vs. 22%, *p =* .008), associated with an increased E/A ratio (1.3 ± 0.4 vs. 1.0 ± 0.3, *p =* .08), than those with a D‐pattern. There were no further differences between the two groups (Table 1). Multivariate regression showed that an advanced age (OR 1.1 per year, CI 1.007–1.199, *p =* .034) and a higher E/A ratio (OR 14.1, CI 1.013–196.1, *p =* .049) were independent predictors of the U‐pattern. In order to mitigate the effect of collinearity we used a separate multivariate model, were diastolic dysfunction (OR 6.36, CI 1.132–35.7, *p =* .036) showed higher significance than the E/A ratio and age (OR 1.08 per year, CI 1.001–1.17, *p =* .047) remained an independent predictor of the U‐pattern (Table 2). Similarly, multiple linear regression analysis revealed that after adjusting for U‐pattern and gender, diastolic dysfunction (B 4.49, CI 1.61–7.37, *p =* .003) remained the only independent predictor of ASI (Table S1).

**TABLE 2:** Univariate and multivariate regression for predictors of a U‐pattern in AF patients in a model (a) with the E/A ratio and a model (b) with the DD

	Univariate analysis			Multivariate model 1			Multivariate model 2		
	OR	CI	*p*	OR	CI	*p*	OR	CI	*p*
Age, /year	1.09	1.013–0.178	.022	1.1	1.007–1.199	.034	1.08	1.001–1.17	.047
E/A, n	7.90	0.719–86.786	.091	14.1	1013–196.1	.049			
DD	8.36	1.582–44.195	.012				6.36	1.132–35.7	.036
ASI, %	1.10	0.981–1.235	.102						
LAV, /ml	1.01	0.987–1.008	.470						

Abbreviations: AF, atrial fibrillation; ASI, asymmetry index of the left atrium; DD; diastolic dysfunction; LAV, left atrial volume.

## 4 DISCUSSION

### 4.1 Main findings

We performed a thorough prospective investigation of atrial conduction times using PW‐TDI and a detailed analysis of anatomical LA changes in AF patients to identify their association. In contrast to previous studies,^6^ we measured the local activation at four LA sites and described two distinct activations: an upward U‐pattern with pronounced delay at the anterior wall and a D‐pattern. The pathologic U‐pattern was associated with an increased LA asymmetry (ASI) and was driven mainly by advanced age and diastolic dysfunction. Diastolic dysfunction was also the only independent predictor of asymmetry (ASI), suggesting a common pathophysiologic pathway of the electromechanic and anatomic LA remodeling.

To the best of our knowledge, this is the first attempt to study the relation of atrial geometry with the mechanical LA activation as measured by PW‐TDI. These findings may help to better understand AF progression and could facilitate patient‐specific ablation strategies.

### 4.2 Atrial remodeling and pathologic LA activation

The present data add to our knowledge by studying the missing links between electrical, mechanical and anatomical LA remodeling in AF patients. In line with our previous results and other recent studies,^8^, ^12^ we found that the echocardiographic PW‐TDI findings had a very good correlation with the intracardiac electrophysiological measurements, supporting the use of PW‐TDI as a simple and reliable surrogate for local electrical activation.

As previously described, the delayed anterior LA activation (upward U‐pattern) represents an impairment of the normal LA activation that is mostly seen in AF patients. Since the BB is the preferential electrical connection between the right atrium and the antero‐superior LA part, a block in this route could change the course of LA activation from a D to a U‐pattern. These results invigorate previous studies showing an association between AF and the interatrial delay (biphasic P wave) caused by deterioration of the BB conduction or more prominent inferiorly located interatrial connections.^13^, ^14^, ^15^ Ablation or pacing at the BB has shown a hampering effect on AF progression, by preventing reentrant circuits that involve the BB.^16^, ^17^, ^18^, ^19^ Therefore, identification of predisposing factors for a BB block or a U‐pattern could help us detect patients that may profit from an earlier or more vigorous rhythm‐therapy. In accordance with the study of Xia et al, our study found that advanced age and diastolic dysfunction (i.e., E/A) were important predisposing factors for a U‐pattern and thus an asynchronous LA contraction.^20^

In the present study, this asynchronous U‐pattern was associated with an increased asymmetry index (65 ± 6% vs. 61 ± 3%, *p =* .01). This represents a prominent increase of the anterior LA and has been previously proven to be an independent predictor for AF recurrence after CA.^1^, ^2^, ^3^

Since ASI was not an independent predictor of the U‐pattern, we hypothesize that these two remodeling surrogates share a parallel pathophysiological process with common causes. This is further supported by our recent findings that emphasize the pathophysiological importance of diastolic dysfunction (LVDD) on the asymmetric LA remodeling.^11^ Similarly, this study found that after adjusting for gender and U‐pattern, the only independent predictor of ASI was LVDD, probably representing a common pathway of the electromechanic and anatomical remodeling.

An increased LV stiffness or a decreased relaxation translates into higher LA pressure with reduced LA emptying and finally atrial dilatation, all promoting electromechanical delay and the risk for AF.^21^, ^22^ Our results complement previous studies and imply that LVDD can result in asymmetric LA dilatation and a specific delayed anterior activation. These characteristics specify advanced AF that might necessitate changes in management to address the developing substrate, for example, radiofrequency ablation instead of cryoablation.^9^, ^10^

Therefore, comprehensive evaluation of the remodeling should take into account variables such as asymmetrical dilatation and changes in activation sequence. These surrogates of remodeling could shed a light in to the complex pathophysiology of the AF, help us improve the stratification of patients and select those for a one‐shot device.

### 4.3 Clinical implications

This study demonstrates that a pathologic electromechanical activation is associated with LA remodeling and that both share the casual pathway of diastolic dysfunction. Since the usual risk factors for AF progression have limited predictive value, the implementation of new and more sensitive predictors could help frame earlier and more appropriate therapeutic decisions. Our study suggests that PW‐TDI echocardiography and the LA asymmetry index (ASI) could reliably represent different stages of the disease in AF patients. Since an increased anterior LA asymmetry and a delayed anterior contraction (U‐pattern) represent typical features of advanced AF, their role in arrhythmogenesis should be further investigated. These results should be seen as hypothesis generating for future studies that may aim to tailor treatment to patient‐specific characteristics, for example, PV isolation for an early AF stadium or more aggressive substrate ablation in the atria of patients with increased ASI or a U‐pattern.

## 5 LIMITATIONS

In order to perform PW‐TDI measurement during sinus rhythm, more patients with paroxysmal AF and without valvular AF were enrolled in this study. Cardioversion prior to ablation and inclusion of more patients with persistent AF may have drawn a different picture. Therefore, this pattern should not be applied to patients with persistent/permanent AF or severe valvular disorders. Although poor acoustic windows, cardiac movements or passive deformation of the adjacent segments could influence the results, repeated PW‐TDI measurements were averaged to mitigate these effects. Despite the similar use of antiarrhythmic drugs in the groups, the study was not powered enough to address the effect of drugs on atrial electrophysiology or the effects of the U‐pattern and ASI on AF recurrences. Although we previously demonstrated the association of the U‐pattern with low‐voltage areas,^9^ the current study was not aiming and was powered to assess the interaction between ASI, LA scar and activation. Our group is working on an automated shape analysis in a larger cohort, but ASI in this study was calculated manually, with good inter and intraobserver agreement, limiting the size of the study. Finally, since impaired LV systolic function, severe hypertrophy and valvular disease were exclusion criteria, the results of this study may not be valid for patients with these conditions. More studies are needed to examine the relation of LA activation and anatomy changes in such patients as selection/intervention criteria.

## 6 CONCLUSION

A delayed activation at the anterior MA in PW‐TDI (U‐pattern) in AF patients is associated with increased LA asymmetry (mostly anterior dilatation). Diastolic dysfunction is a common cause of this pathologic activation and remodeling. Therefore, detection of a U‐pattern signifies advanced AF stages and may facilitate selection for an appropriate ablation strategy (radiofrequency) than can address additional substrate.

## CONFLICT OF INTEREST

The authors declare no potential conflict of interest.

## Supporting information


**Table S1** Linear regression analysis for predictors of LA asymmetry (ASI).

## Data Availability

Data are available upon reasonable request from the corresponding author.
