# Supplementary material for: Left atrial activation and asymmetric anatomical remodeling in patients with atrial fibrillation: The relation between anatomy and function
Source: Clin Cardiol. 2020 Nov 17;44(1):116–22. doi: 10.1002/clc.23515 (PMC7803371; doi:10.1002/clc.23515)
Supplement: Supplementary file 1 — Table S1 Linear regression analysis for predictors of LA asymmetry (ASI). [file CLC-44-116-s001.doc]

**Supplementary Table 1.** Linear regression analysis for predictors of LA asymmetry (ASI)

|  | **Univariate analysis** | | | **Multivariate analysis** | | |
| --- | --- | --- | --- | --- | --- | --- |
| **Variables** | **B** | **95% CI** | **P** | **B** | **95% CI** | **P** |
| Age, years | 0.05 | -0.08, 0.18 | 0.46 |  |  |  |
| BMI, n | -0.25 | -0.69, 0.19 | 0.26 |  |  |  |
| Female, n (%) | 2.48 | -0.35, 5.29 | **0.08** | 2.12 | -0.72, 4.97 | 0.22 |
| Heart failure, n (%) | -0.72 | -5.65, 4.19 | 0.77 |  |  |  |
| Coronary/vascular disease, n (%) | 2.96 | -2.28, 8.19 | 0.26 |  |  |  |
| Diabetes, n (%) | 2.12 | -2.06, 6.31 | 0.32 |  |  |  |
| Hypertension, n (%) | -0.56 | -3.79, 2.67 | 0.73 |  |  |  |
| Persistent atrial fibrillation, n (%) | -0.58 | -3.99, 2.84 | 0.74 |  |  |  |
| Beta-blockers, n (%) | 0.031 | -3.74, 4.35 | 0.88 |  |  |  |
| Antiarrhythmic drugs Cl. I/III, n (%) | 1.09 | -1.91, 4.09 | 0.47 |  |  |  |
| **Echocardiography** |  |  |  |  |  |  |
| LV ejection fraction, mm | 0.84 | -1.32, 0.03 | 0.44 |  |  |  |
| Inter-ventricular septum, mm | -0.22 | -0.88, 0.44 | 0.51 |  |  |  |
| LV end diastolic diameter, mm | -0.19 | -0.27, 0.23 | 0.88 |  |  |  |
| LA diameter, mm | -0.18 | -0.44, 0.07 | 0.16 |  |  |  |
| LA area index, mm/m^2^ | -0.51 | -0.91, 1.78 | 0.65 |  |  |  |
| LA emptying fraction, % | -0.05 | -0.19, 0.09 | 0.48 |  |  |  |
| E/A, n | 1.25 | -1.69, 4.21 | 0.39 |  |  |  |
| Deceleration time, ms | -0.17 | -0.05, 0.01 | 0.25 |  |  |  |
| Diastolic dysfunction, n (%) | 4.49 | 1.61, 7.37 | 0.003 | 4.49 | 1.61, 7.37 | 0.003 |
| Mitral annulus P-A’ septal, ms | -0.05 | -0.12, 0.03 | 0.21 |  |  |  |
| Mitral annulus P-A’ lateral, ms | -0.03 | -0.09, 0.03 | 0.29 |  |  |  |
| Mitral annulus P-A’ posterior, ms | -0.02 | -0.09, 0.06 | 0.63 |  |  |  |
| Mitral annulus P-A’ anterior, ms | 0.01 | -0.06, 0.07 | 0.87 |  |  |  |
| P-CS 9-10, ms | -0.01 | -0.09, 0.09 | 0.96 |  |  |  |
| P-CS 1-2, ms | 0.02 | -0.08, 0.12 | 0.66 |  |  |  |
| U-Pattern | -3.77 | -8.0, 0.65 | **0.09** | 3.12 | -1.23, 7.47 | 0.08 |
| **Computed tomography** |  |  |  |  |  |  |
| LA volume, ml | -0.01 | -0.1, 0.04 | 0.77 |  |  |  |
| LA anterior volume, ml | -0.24 | -0.3, -0.15 | <0.001 |  |  |  |
| LA posterior volume, ml | 0.08 | 0.02, 0.15 | 0.017 |  |  |  |

BMI = Body Mass Index, CL. = classm LA = left atrial, LV = left ventricular
